# Undifferentiated Adenocarcinoma With Rhabdoid Features of the Stomach: A Case Report

**DOI:** 10.7759/cureus.87321

**Published:** 2025-07-05

**Authors:** Soufiane Taibi, Ouryemchi Mouad, Haitam Soussan, Guellil Abdelali, Mohammed Bouziane

**Affiliations:** 1 Department of General Surgery, Mohammed VI University Hospital, Faculty of Medicine and Pharmacy, Laboratory of Anatomy, Microsurgery and Surgery Experimental and Medical Simulation (LAMCESM), Oujda, MAR; 2 General Surgery, Centre Hospitalier Universitaire Mohammed VI, Oujda, MAR

**Keywords:** adenocarcinoma, cytokeratin, ini1 gene, rhabdoid tumor, vimentine

## Abstract

Rhabdoid tumors are rare and aggressive neoplasms, first described in the kidney, but they have also been reported in various extrarenal sites, including the gastrointestinal tract. Gastric involvement is particularly unusual, often presenting as an adenocarcinoma with rhabdoid features. This type of tumor poses a diagnostic challenge, requiring histological, immunohistochemical, and cytogenetic analyses. Due to the aggressive nature of these tumors, early diagnosis and prompt surgical intervention are essential, although the prognosis remains poor. We report a case of a man with an upper gastrointestinal bleeding where both gastrointestinal endoscopy, colonoscopy, and even the abdominal scan failed to identify the origin of bleeding; considering these results, a Weinberg procedure was performed. In light of the continued deterioration, antrectomy was deemed necessary, after which anatomopathological findings were consistent with an undifferentiated adenocarcinoma of the stomach exhibiting rhabdoid features.

## Introduction

Rhabdoid tumors are a distinct group of neoplasms reported in renal and extrarenal sites that were first described by Beckwith and Palmer in 1971 and initially observed in the kidneys of children [1]. Among these extrarenal sites, the gastrointestinal tract is a notable location. However, cases of gastric adenocarcinoma with rhabdoid features remain rare, with only a few documented cases in the literature [2]. Recognition of the rhabdoid phenotype in gastrointestinal neoplasms, based on morphological, immunohistochemical, and cytogenetic features, is essential due to its association with poor prognosis and resistance to conventional therapies [3]. We present a rare and aggressive variant of gastric cancer, undifferentiated adenocarcinoma with rhabdoid features, to underscore the diagnostic and therapeutic challenges through a clinical case.

## Case presentation

**Figure 1:**
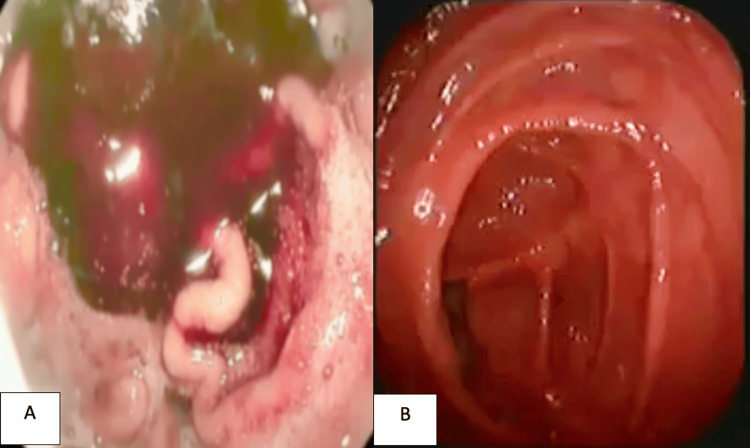
A. Gastroduodenal endoscopy image shows blood stasis without any actual bleeding. B. Colonoscopic image shows no abnormality.

**Figure 2:**
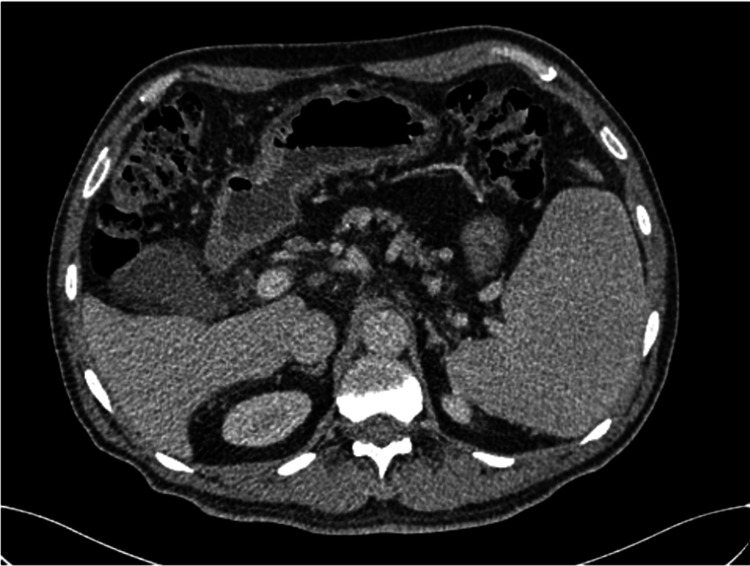
CT scan image shows no abdnormality in the stomach.

A 69-year-old male with a history of alcoholism and chronic smoking presented to the emergency department with moderate upper gastrointestinal hemorrhage, represented by two episodes of hematemesis and melaena evolving more than six hours before his admission, without any other signs, in the context of deteriorated general health, evidenced by a 6 kg weight loss over one month. The patient was conscious and exhibited stable vital signs, including hemodynamic and respiratory stability; the abdominal examination was unremarkable, no splenomegaly or adenopathy was found on palpation, and the rectal examination showed melaena. Laboratory tests indicated hypochromic anemia (hemoglobin (Hb) of 5.5 g/dl) (reference range: 13-18 g/dL). The rest of the laboratory tests were normal. However, a gastroduodenal endoscopy was performed where fresh red blood stasis was found in the stomach without noticing any active bleeding; in addition, a colonoscopy showed no abnormalities, and an abdominal CT scan was normal as well (Figures 1-2).

**Figure 3:**
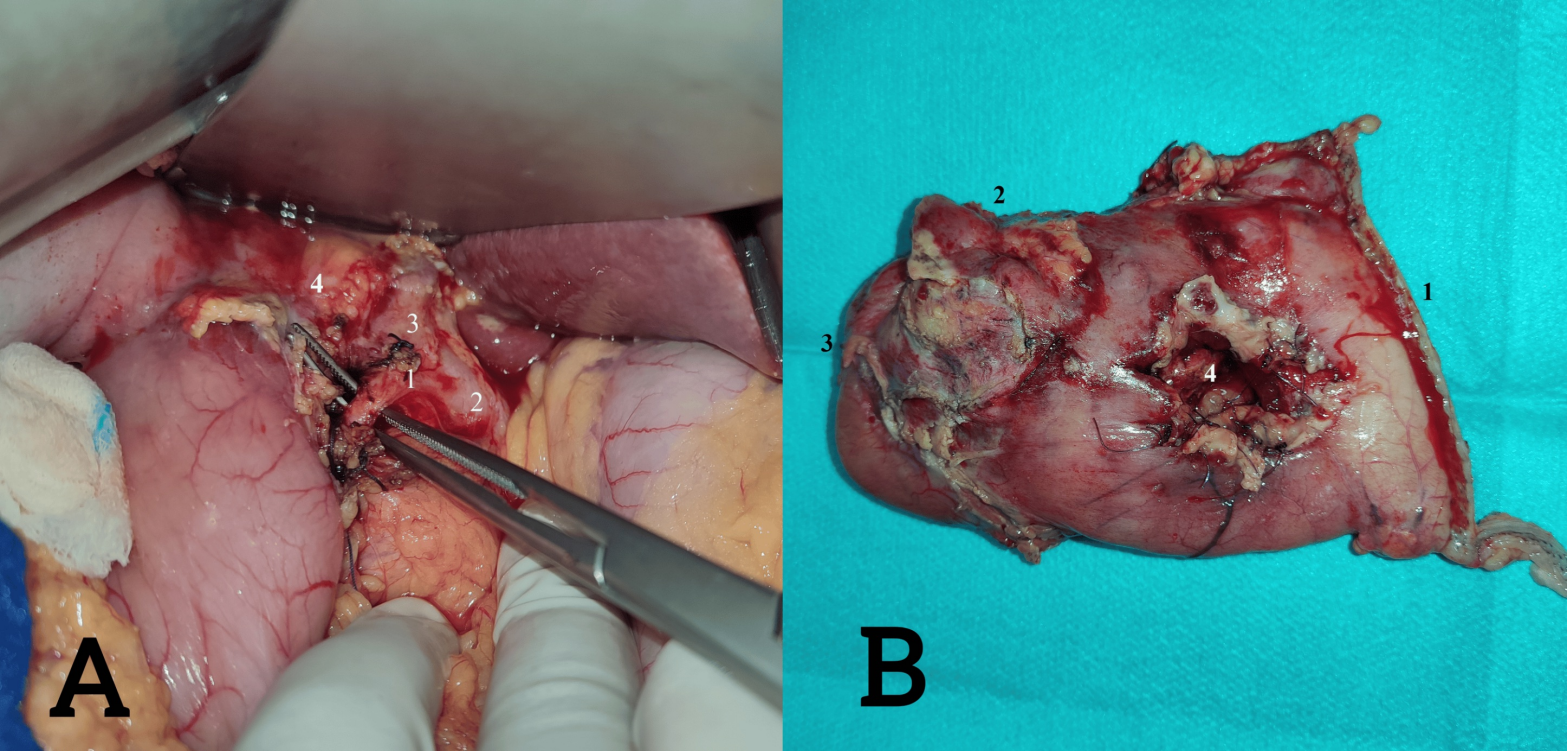
A. Intraoperative image during identification of the gastroduodenal artery for the Weinberg procedure. B. Image showing the antrectomy specimen following the failure of the Weinberg procedure. Image A: 1. gastroduodenal artery, 2. common hepatic artery, 3. proper hepatic artery, 4. common bile duct. Image B: 1. gastric resection margin, 2. duodenal resection margin, 3. duodenal bulb, 4. previous duodenotomy from the Weinberg procedure.

Despite transfusion, the patient’s clinical and biological condition did not improve, prompting the decision to perform an exploratory laparotomy with a Weinberg procedure. One day later, our patient kept low hemoglobin figures even after transfusion; the patient’s case was presented in a multidisciplinary meeting, in the presence of a gastroenterologist, radiologist, and general surgeons, where a hemostasis antrectomy decision was taken. After performing the antrectomy with gastrojejunal anastomosis, the postoperative follow-up was uneventful without any particular incidents, and the hemoglobin level was stable (Figure 3).

**Figure 4:**
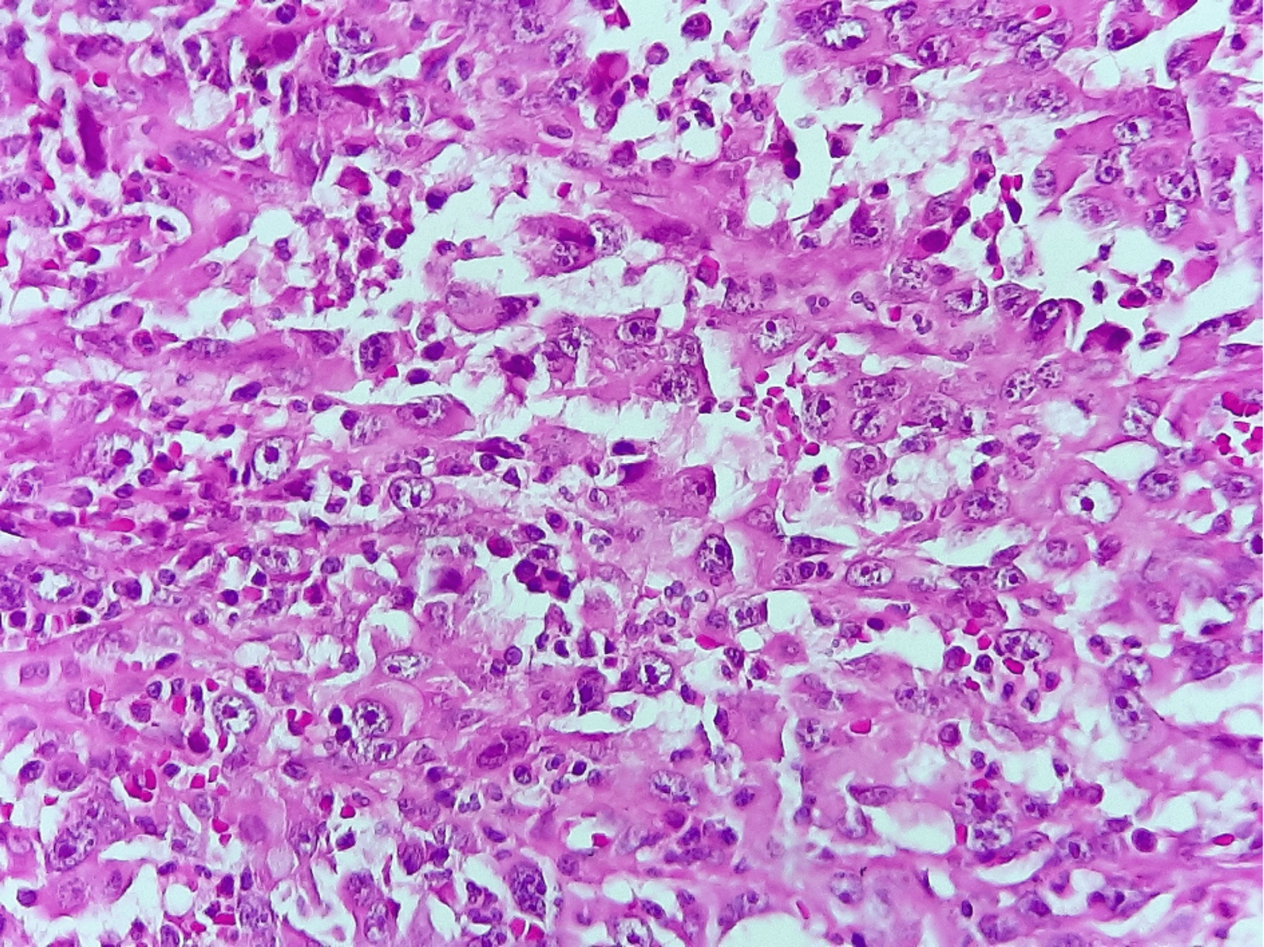
Histological examination x400. Tumor cells shows atypie, multinucleation, and marked pleomorphism.

Histological examination of the resected specimen reveals a proliferation of highly atypical cells arranged in diffuse sheets. No glandular formations were seen. Most tumor cells show evident atypia with marked pleomorphism and multinucleation. Nuclei are enlarged, irregular, and contain prominent nucleoli. Several mitotic figures were observed (Figure 4).

**Figure 5:**
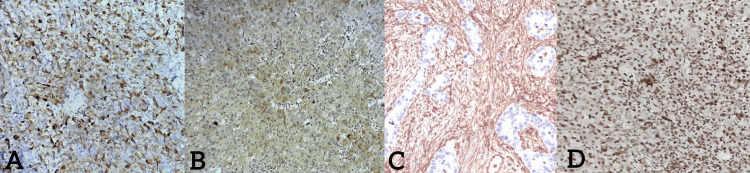
Immunohistochemical staining shows a positivity for cytokeratin (A), epithelial membrane antigen (EMA) (B), and vimentin (C) while cytogenetic staining shows positivity for the integrase interactor (INI1) mutation (D).

Immunohistochemical staining shows positivity for cytokeratin, epithelial membrane antigen (EMA), and vimentin. Other differentiation markers were all negative. As a complement to a cytogenetic study, the integrase interactor (INI1) mutation was found positive in this case (Figure 5). Given these findings, the diagnosis of undifferentiated adenocarcinoma with rhabdoid features of the stomach was made, and the patient was referred to the oncology department for further management.

## Discussion

Gastric cancer remains a significant clinical and diagnostic challenge. While tubular adenocarcinoma is the most common form of stomach cancer, rare variants, such as undifferentiated adenocarcinoma with rhabdoid features, are still poorly understood. Sommers and Meissner first described these tumors in 1954 as "pleomorphic giant cell carcinoma" of the pancreas. Since then, similar tumors have been identified in other organs, including the stomach, under the same designation [4]. This localization, however, is exceptionally rare. For instance, Shomori et al. reported that only 16 cases have been documented [5], while Ueyama et al. found that only four out of 5,437 cases of gastric adenocarcinoma exhibited rhabdoid features [6]. The present case aligns with these reports, further emphasizing the rarity of this tumor type in gastric localization. Our findings are consistent with previous descriptions of rhabdoid tumors presenting in extrarenal sites such as the esophagus, small intestine, large intestine, liver, and pancreas, all of which share the histological hallmark of adenocarcinomas with rhabdoid features [7]. This case adds to the small body of documented evidence by reinforcing the notion that rhabdoid differentiation can occur within undifferentiated gastric carcinomas and may manifest clinically with nonspecific symptoms, as in our patient who presented with gastrointestinal bleeding and weight loss - features that are not unique to rhabdoid tumors but reflect their aggressive nature.

The emergence of rhabdoid tumors has been linked to chromosomal abnormalities, particularly monosomy 22 and structural alterations involving the 22q11.2 region. Through positional cloning, researchers identified the SMARCB1 gene - also known as INI1, SNF5, or BAF47 - as a key factor in the pathogenesis of these tumors. SMARCB1, part of the SWI/SNF chromatin remodeling complex, functions as a tumor suppressor. Germline deletions or mutations in this gene increase susceptibility to rhabdoid tumors, while somatic alterations in the remaining allele represent the "second hit" in tumor development. The complete loss of SMARCB1 protein expression, typically observed in the cell nucleus, can be confirmed via immunohistochemical analysis [8]. Similar to previously reported cases, the immunohistochemical profile in our patient demonstrated loss of INI1 expression, a diagnostic hallmark of rhabdoid tumors, thereby confirming the diagnosis at the molecular level. These tumors are more commonly observed in older male adults, and, in many cases, they present with an atypical clinical course, often lacking the characteristic symptoms typically associated with these malignancies, which can complicate early diagnosis and clinical management [9]. Up to now, little is known about the imaging findings. However, existing case reports describe heterogeneous tumors with necrosis, gastric wall thickening, and metastases to lymph nodes, liver, and pancreas, often resembling advanced gastric cancer or lymphoma on imaging but with distinctive features such as contrast enhancement and infiltration into surrounding tissues helps differentiate this kind of tumor [10]. Our experience supports the assertion that imaging may not be definitive in differentiating rhabdoid tumors from other aggressive gastric malignancies.

Diagnosis is established through a combination of histological evaluation, immunohistochemical staining, and cytogenetic analysis. At low magnification, the tumor's surface may resemble a moderately differentiated tubular adenocarcinoma. However, higher magnification reveals solid areas composed of tumor cells with vesicular nuclei and a cytoplasm that is abundantly eosinophilic [5]. The most immunohistochemical markers found positive were vimentin, which occurs in mesenchymal cells, and cytokeratin in epithelial cells; the expression, including these two factors, appears restricted to relatively few tumor types, as in our case [6]. Vimentin positivity in gastric cancer suggests a highly invasive phenotype and may play a crucial role in the metastasis of gastric carcinoma [11]. In addition, positive vimentin expression could serve as a poor prognostic marker in gastric cancer [12]. Cytogenetic diagnosis of these tumors typically involves identifying mutations in the INI1 gene, as already described above [13].

This case not only mirrors the clinical presentation, histopathological features, immunohistochemical profile, and molecular alterations described in previous reports but also contributes to the limited number of documented cases in the literature. By doing so, it expands the current understanding of this rare and aggressive tumor subtype and underscores the critical importance of including undifferentiated gastric adenocarcinoma with rhabdoid features in the differential diagnosis when encountering poorly differentiated gastric neoplasms.

## Conclusions

The diagnosis of gastric adenocarcinoma with rhabdoid features is confirmed through a combination of histological, immunohistochemical, and cytogenetic studies. The identification of mutations in the INI1 gene, which is associated with these rare tumors, is critical for accurate diagnosis and understanding of the underlying molecular mechanisms. Further research is needed to better characterize the imaging, clinical features, and molecular profiles of these rare gastric neoplasms, which may lead to more effective diagnostic and therapeutic strategies.
